# Graphene-Wrapped ZnO Nanocomposite with Enhanced Room-Temperature Photo-Activated Toluene Sensing Properties

**DOI:** 10.3390/ma17051009

**Published:** 2024-02-22

**Authors:** Qingwu Huang, Jinjin Wu, Dawen Zeng, Peng Zhou

**Affiliations:** 1 Analytical and Testing Center of Huazhong University of Science and Technology, Huazhong University of Science and Technology, No. 1037, Luoyu Road, Wuhan 430074, China; 2 Nanomaterials and Smart Sensors Laboratory (NSSL), Department of Materials Science and Engineering, Huazhong University of Science and Technology, No. 1037, Luoyu Road, Wuhan 430074, China; 3 State Key Laboratory of Materials Processing and Die & Mould Technology, Huazhong University of Science and Technology, No. 1037, Luoyu Road, Wuhan 430074, China

**Keywords:** graphene, ZnO, photo-activated, room temperature, gas sensing, toluene, solvothermal technology

## Abstract

Graphene-wrapped ZnO nanocomposites were fabricated by a simple solvothermal technology with a one-pot route. The structure and morphology of these as-fabricated samples were systematically characterized. The adding of graphene enhanced the content of the oxygen vacancy defect of the sample. All gas-sensing performances of sensors based on as-prepared samples were thoroughly studied. Sensors displayed an ultrahigh response and exceptional selectivity at room temperature under blue light irradiation. This excellent and enhanced toluene gas-sensing property was principally attributed to the synergistic impacts of the oxygen vacancy defect and the wrapped graphene in the composite sensor. The photo-activated graphene-wrapped ZnO sensor illustrated potential application in the practical detection of low concentrations of toluene under explosive environments.

## 1. Introduction

As an emerging and multidisciplinary science, nanotechnology is based on the design and application of nanostructures. With the ability to control matter at the molecular or even atomic level (1–100 nm), nanostructures exhibit many interesting properties, such as size, morphology, and their area–volume relationship. All these characteristics allow nanostructures significant potential application in gas-sensing detection processes. Considering their high sensitivity, low cost, and excellent stability, infrastructure metal oxide semiconductor (MOX) gas-sensing materials, such as ZnO, WO_3_, and SnO_2_, have been widely researched in relation to detecting industrial gases, respiratory system gas analysis, and indoor air pollutants [1,2]. However, conventional MOX gas sensors usually work at a high operating temperature, typically above 200 °C. A high temperature also brings many limitations and problems to thermally excited gas sensors. Firstly, a high working temperature accelerates the passivation of oxide semiconductor sensitive materials, which has adverse effects on maintaining a device’s lifespan and detection consistency. Secondly, the existence of heating devices is unfavorable for device integration, power consumption control, and deteriorates the reliability and suitability of the sensor device [3,4]. In addition, a high working temperature can pose a risk of explosion, and limits application conditions, such as those associated with flammable, hazardous, and explosive gas detection. Many efforts have been made in this field to decrease the working temperature. In order to maintain the low-temperature working ability of gas sensors, light energy has been used to provide sensors with external energy. In recent reports, MOX-based gas sensors have shown to detect target gases under photon activation [5,6,7].

It was reported by Gulyaev et al. that a SnO_2_ thermal excitation sensor irradiated with a LED light source improved the selectivity of the sensor to ethanol [7]. Gu et al. reported a WS_2_ light-assisted gas sensor irradiated by LEDs that was powered by a nano friction generator. Sensors realized a gas-sensing response at near room temperature (40 °C) under UV, visible light, and near-infrared irradiation [8]. Cui et al. prepared gas sensors based on ZnO with different nanostructures and tested their HCHO gas-sensing performances at room temperature under the irradiation of 365 nm UV light. Results showed that gas sensors based on ZnO nanofibers demonstrated a reversible response, high sensitivity, and good selectivity [9]. However, UV lights still consumed a significant amount of energy and the price was high. Visible low-cost, energy-saving light should be used instead of UV light.

Functionalized nanoscale ZnO with metal nanoparticles, such as Pd and Pt nanoparticles [10,11], has been applied to test H_2_ and NH_3_ gases at room temperature. However, its sensing sensitivity and response were low, and did not meet the requirements of practical applications for monitoring environmental gas pollution. In addition, due to the selective catalytic activity of the surface’s functionalization, the types of gases that could be detected were limited. As a monolayer atom of two-dimensional carbon materials, graphene has shown gas sensitivity properties to gas molecules at room temperature [12]. For instance, graphene exhibits a sensing ability for NH_3_ [13] at room temperature due to the variation in its electrical resistance signal when exposed to these detecting gases. Different from other carbon materials, graphene demonstrates an inherently low electrical noise [12], large surface area, ultrahigh electron mobility [14,15], and ballistic charge carrier transport [16]. All of these properties are clear requirements for optimal electrically-based gas sensors that are sensitive to the adsorption of gas molecules. However, experimental and theoretical [17] research has shown that detection gas molecules show feeble adsorption on the original graphene surface, which is far from the requirements of actual environmental, safety, or industrial detection. Many technical routes have been developed to enhance the absorption of the detecting gas on the graphene, such as functionalization by doping, decorating, or hybridizing with other materials [18,19,20,21]. Shafiei et al. [22] reported that when decorated with metal Pt or Pd, graphene sensors demonstrated obvious H_2_-sensing properties at room temperature. Surface-functionalized graphene [23] or small metal oxide quantum dots used to decorate graphene [24] have also been reported to enhance gas-sensing properties.

Here, we report toluene sensors based on graphene-wrapped ZnO nanocomposites with enhanced performance at room temperature. Nanosized samples were fabricated by a simple solution treatment method that had the characteristics of easy operation, controllable particle size, low energy consumption, and inexpensive equipment requirements. The graphene and ZnO nanoparticles demonstrated a synergistic effect that caused enhanced gas-sensing properties compared to those of a pure ZnO sensor. These graphene-wrapped ZnO nanocomposite sensors showed good selectivity to toluene gas and worked well under a high humidity environment at room temperature. The gas sensors’ mechanism is discussed in detail herein.

## 2. Experimental

In a typical experimental process, sodium (3 g, >99.7 wt.% pure), zinc nitrate hydrate (3 g, >99.999 wt.% pure) and fructose (3 g) with absolute ethyl alcohol (30 mL) as a solvent were reacted at 220 °C for 72 h in a Teflon-lined sealed reactor vessel (Parr Instr. Cor., Champaign, IL, USA, 4843) based on previous reports [24,25]. The gray precursor was rapidly pyrolysed at 300 °C in a muffle furnace under nitrogen atmosphere protection. The obtained black powder was thoroughly washed by de-ionized water and methyl alcohol several times, then filtered and dried in a vacuum. The product was also fabricated without adding fructose and sodium for comparison. For clearance, the as-prepared products were named G-ZnO and pure ZnO, respectively.

**Figure 1:**
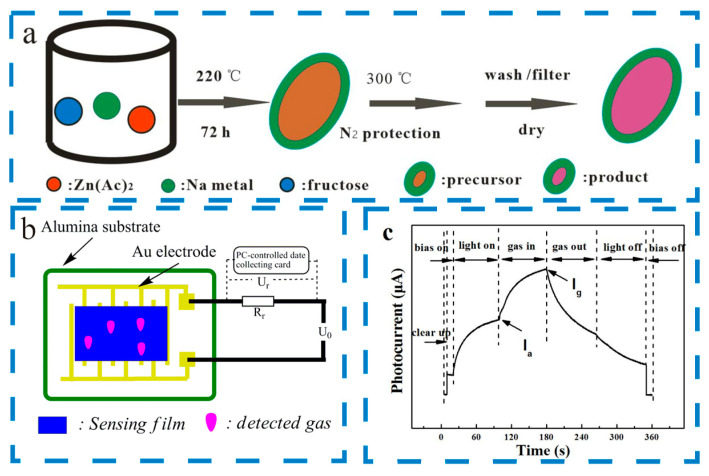
The schematic illustration of the fabrication process of gas-sensing materials (**a**), the detailed electrode configuration and gas sensor structure (**b**), and the procedure for photo-activated gas sensor testing and typical test curve (**c**).

Samples were characterized by Raman spectroscopy (Horiba Jobin Yvon Company, Paris, France, LabRAM HR800), X-ray photoelectron spectroscopy (XPS) (Kratos Company, Manchester, UK, Axis Ultra DLD), X-ray diffraction (XRD) (Philips Company, Amsterdam, The Netherlands, X’Pert Pro), scanning electron microscopy (SEM) (FEI Company, Hillsboro, OH, USA, Sirion 200 FE-SEM), transmission electron microscopy (TEM) (JEOL Company, Tokyo, Japan, JEM-2100F STEM), and electron paramagnetic resonance (EPR) (BRUKER Company, Karlsruhe, Germany, EPR-E500). Photo-activated gas-sensing property testing was conducted on the solid film, partially based on our previously reported work [24]. Sensor devices were fabricated as followed. The as-prepared products (20 mg) were mixed with alcohol (2 mL) to prepare a powder suspension. And the dispersion solution was then gently released onto the gold interdigital electrode. The Au electrode was preprinted on the Al_2_O_3_ substrate. In order to improve the contact and its stability, all sensor devices were annealed under vacuum at 180 °C. The test platform was cleaned by dry air flow for 10 min before testing, and used a flat blue LED array light source (475 nm, 80 W/m^2^) with a 5 V bias voltage at 25 °C. The whole test process lasted 360 s, and was divided into the following stages: 0~5 s was the initial state stage of the test system; 5–10 s was the zero clearing stage of the test system; bias voltage was applied at 10 s; the light source was turned on at 20 s; the detecting gas was introduced into the detecting chamber at 100 s; the detecting gas was off at 180 s; the light source was turned off at 265 s; the bias voltage was turned off at 350 s, and the testing process ended at 360 s. The dry air and detecting gas were controlled by a gas flow meter, and photocurrent responses were recorded by a computer-controlled data collecting card (6008, National Instruments Cor., Austin, TX, USA). The gas flow meter and data collecting card were controlled by a computer program. Photo-activated gas sensors’ performances were calculated by photocurrent responses. The response value (Response, R) is defined as R = (Ig − Ia)/Ia, where Ia is the photocurrent before introducing detecting gas and Ig is the photocurrent after introducing detecting gas. Figure 1a shows a schematic illustration of the fabrication process for gas-sensing materials, and Figure 1b shows the detailed electrode configuration, the gas sensor’s structure, the procedure for photo-activated gas sensor testing, and a typical test curve.

## 3. Results and Discussion

**Figure 2:**
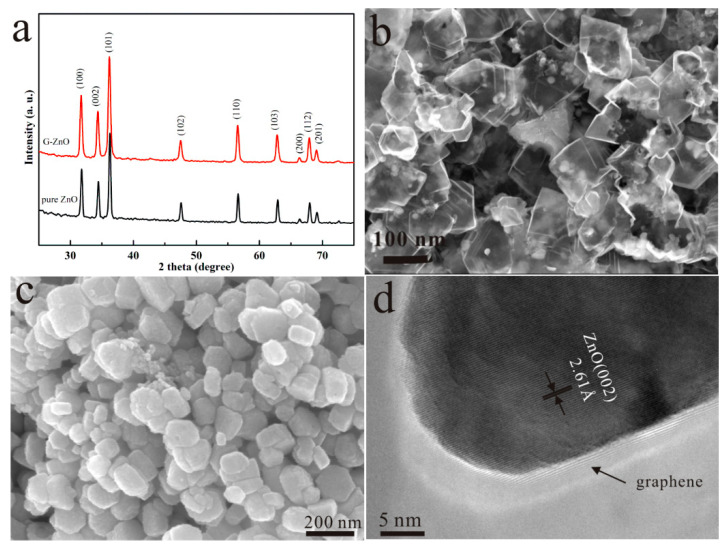
XRD spectra (**a**) of G-ZnO and pure ZnO, SEM image (**b**) and HRTEM image (**d**) of G-ZnO, SEM image (**c**) of pure ZnO.

XRD patterns showed a hexagonal wurtzite structure (International Center for Diffraction Data (ICDD) 36-1452) of pure ZnO and G-ZnO samples, as shown in Figure 2a. Obvious diffraction peaks demonstrated that all samples were well crystallized. For the low content and low atomic number element of graphene, diffraction peaks of graphene were not detected, as previously reported by our group [24]. Figure 2b,c are typical FESEM images of G-ZnO and pure ZnO samples. As reported previously in the literature [25], fructose can be transformed to graphene material by sodium under a reducing atmosphere. And the graphene was totally wrapped onto ZnO nanoparticles with an average diameter of 35.93 ± 11.49 nm, as shown in Figure 2b. Without graphene, the size of pure ZnO nanoparticles increased, with an average diameter of 86.03 ± 9.67 nm, as shown in Figure 2c. The particle size statistical method referred to the report of Francisco et al. [26]. The HRTEM image in Figure 2d shows the edge of silk-like graphene. With an interplanar distance of 0.261 nm, as shown in Figure 2d, the lattice fringe was assigned to the corresponding (002) plane of the hexagonal ZnO.

**Figure 3:**
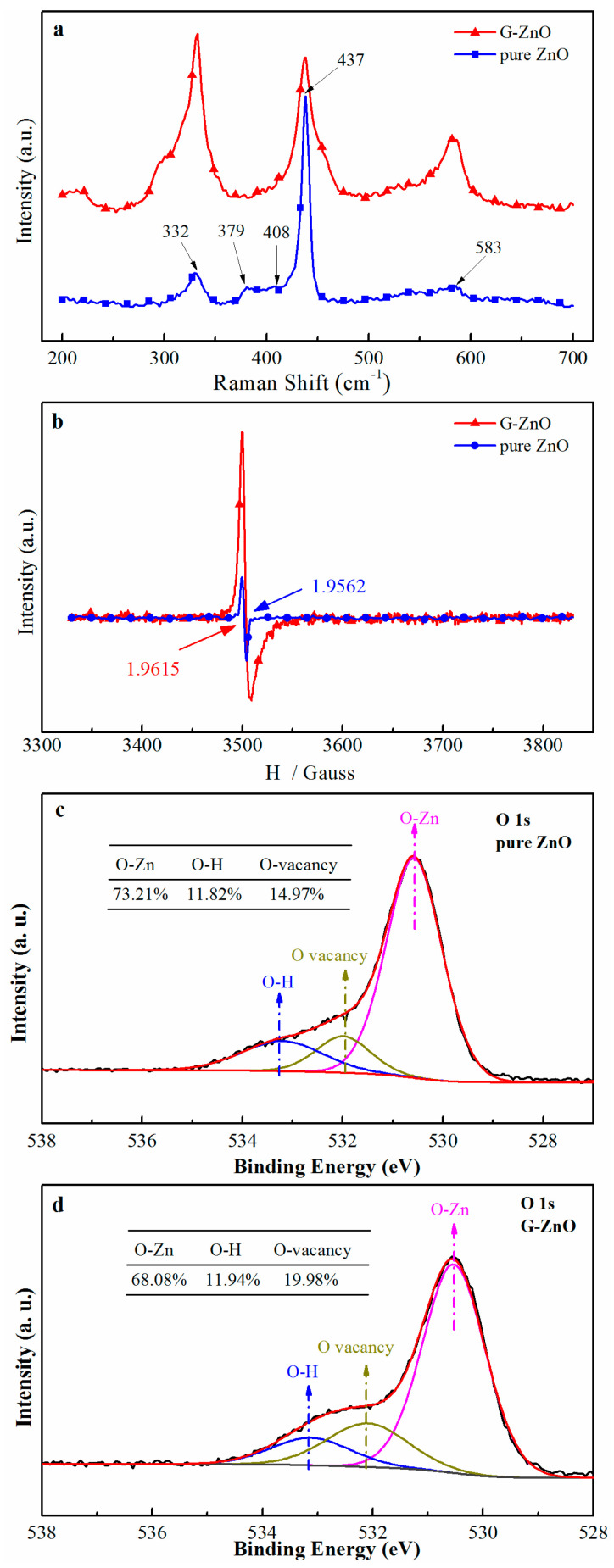
Raman (**a**), EPR (**b**), and XPS (**c**,**d**) spectra of G-ZnO and pure ZnO. The inserts in (**c**,**d**) are quantitative calculation results of O 1s based on fitting peaks.

The Raman spectra of as-prepared samples are shown in Figure 3a. For the pure ZnO sample, the characteristic E2(H) mode peak, E1(LO) mode peak, A1(TO) mode peak, and multiple phonons scattering peak were 437, 583, 379, and 332 cm^−1^, respectively. For the G-ZnO sample, all the peaks at 583 and 332 cm^−1^ become broadened and the intensity increase. Those results indicate that the wrapped graphene could increase the defect in ZnO particles as reported [27,28,29]. This deduction was confirmed by the EPR result shown in Figure 3b. The g values for pure ZnO and G-ZnO were 1.9562 and 1.9615, respectively, which were consistent with other reports, and can be considered as oxygen vacancy defects in ZnO, as reported [30,31]. Importantly, the peak intensity of G-ZnO was about three times that of pure ZnO, which indicated a higher concentration of oxygen vacancy defects in the G-ZnO sample.

The chemical states of oxygen were surveyed by XPS in order to obtain more insights into the oxygen vacancy defects of G-ZnO and pure ZnO materials. As shown in Figure 3c,d, in both samples the XPS spectra of O 1s fitting peaks, located at 530.4–530.6, 531.9–532.1, and 533.4–533.5 eV, could be attributed to lattice oxygen (O lat) in the crystal, oxygen vacancy defects on the surface, and loosely adsorbed oxygen species, such as OH, respectively [29,32]. The content of an oxygen vacancy defect can typically be calculated by the ratio of the area of the fitting peak, and the calculated values were 21.98% and 14.97% in the G-ZnO and pure ZnO samples, respectively. This demonstrates that oxygen vacancies in G-ZnO samples were much higher compared with those of pure ZnO. Obviously, the result of the oxygen vacancy defect as shown by XPS was consistent with the early conclusion of Raman and EPR results.

Based on the above results, graphene-wrapped ZnO nanocomposite was successfully synthesized by a simple one-step solvothermal method. During our fabrication process, active sodium can reduce fructose to graphene. And the strong reducing atmosphere can also cause deoxygenation of ZnO with a higher oxygen vacancy defect content, which was systematically characterized by the test results of Raman, EPR, and XPS. 

**Figure 4:**
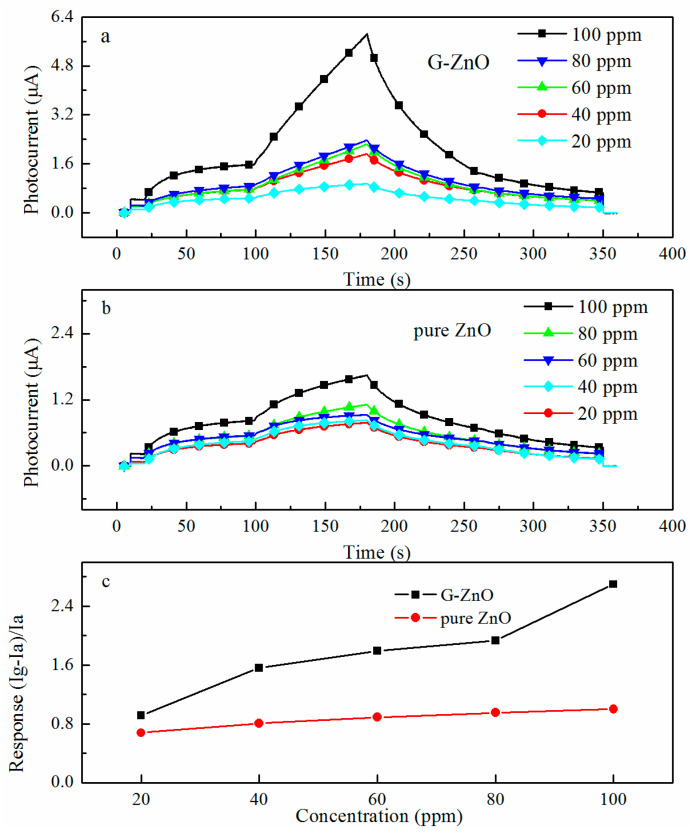
Dynamic response curves of G-ZnO at 20, 40, 60, 80, and 100 ppm toluene (**a**), dynamic response curves of pure ZnO at 20, 40, 60, 80, and 100 ppm toluene (**b**), and response values of G-ZnO and pure ZnO at 20, 40, 60, 80, and 100 ppm toluene, respectively (**c**).

As these samples contained a tunable amount of oxygen vacancy defect, their photo-activated gas-sensing characteristics were expected to be different. As shown in Figure 4a,b, the dynamic sensing response of the two sensors to 100 ppm toluene was measured under blue light irradiation, and the corresponding responses were plotted against different toluene concentrations. The response of G-ZnO nanocomposites to toluene increased from 0.91 to 2.7 as the toluene concentration was enhanced from 20 ppm to 100 ppm. In contrast, the sensing response of pure ZnO nanocomposites to toluene increased from 0.68 to 1.0 as the toluene concentration was enhanced from 20 ppm to 100 ppm. Evidently, the response of G-ZnO nanocomposites to 100 ppm toluene was nearly 2.7 times larger than that of pure ZnO. So, the G-ZnO sensor showed remarkably enhanced photo-activated sensitivity to toluene at room temperature compared with the pure ZnO sensor.

**Figure 5:**
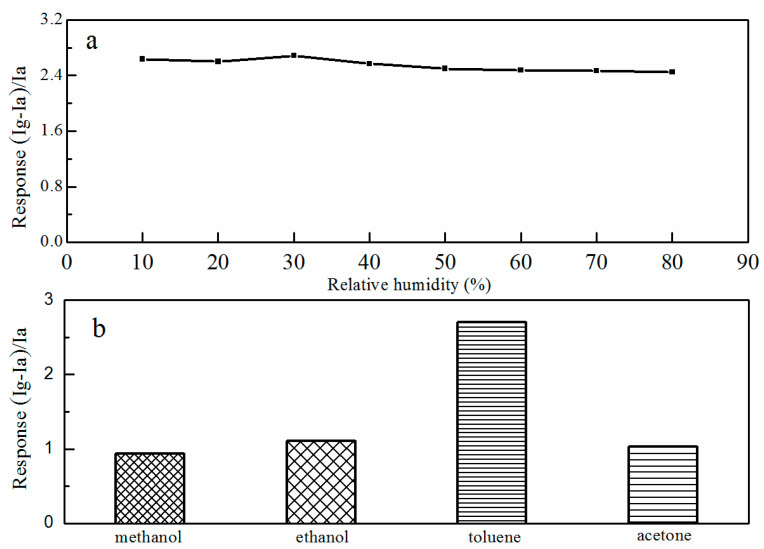
Sensing response of G-ZnO to 100 ppm toluene at different RH levels (**a**) and sensing response of G-ZnO to 100 ppm of different VOCs (**b**).

The sensing response of the G-ZnO sensor under different relative humidity (RH) conditions to 100 ppm toluene was also tested, as shown in Figure 5a. Results showed that at the RH range of 10–30% the gas-sensing response slightly increased. At the RH range of 30–80% the gas-sensing response slightly decreased. This decrease can be explained by the competition between the adsorbed water molecules and the chemisorbed oxygen species on the sensor surface. However, the gas-sensing response decreased slightly under a high humidity condition. This indicated that, even under a high humidity environment, the as-prepared gas sensor could operate effectively.

In practical applications, the selectivity of a gas sensor is important. The gas-sensing response values of the G-ZnO sensor to 100 ppm of methanol, ethanol, toluene, and acetone are shown in Figure 5b. These response values were 0.94, 1.11, 2.7, and 1.03, respectively. Obviously, the G-ZnO sensor had the best selectivity to toluene. Compared with previous reports regarding toluene sensors [33,34,35,36,37,38], which are shown in Table 1, the as-prepared G-ZnO sensor device exhibited a high response and an excellent toluene-sensing performance.

The response mechanism of thermally excited gas sensors is often explained by the electron depletion layer theory [39]. The carrier concentration on the surface of the material can be changed when the gas is adsorbed on the surface of the sensitive material layer. This change is reflected by the resistance of the material, and the gas-sensing response is produced. Different from thermally excited gas sensors, the gas-sensing reaction of photo-activated gas sensors depends on photo-generated electron/hole pairs generated by light irradiation [40]. Taking an n-type semiconductor as an example, when there is no light, electrons on the surface of the material combine with oxygen molecules in the air, which depletes the surface electrons of the material, and then form an electron depletion layer. When the appropriate light irradiates the surface of the material, valence band electrons excite to the conduction band. This excitation produces a large number of electron/hole pairs, and reduces the material’s overall resistance [24]. After introducing the detecting gas, the oxidizing/reducing gas also adsorbs onto the material’s surface, and gains/loses electrons in the adsorption process. This electron transfer affects the width of the electron depletion layer and eventually leads to a decrease/increase in the resistance of the sensitive layer.

Here, the commonly used redox reaction mechanism can explain the gas-sensing mechanism of the G-ZnO gas sensor [41]. In the redox reaction gas-sensing mechanism, chemical oxygen adsorbates play a critical role. Typically, chemical oxygen adsorbates (such as O^−^ or O^2−^) generate on the ZnO surface at an elevated temperature (>100 °C). However, O_2_^−^ adsorbates ions can also be produced on the ZnO surface with a small particle size or under photo-activated condition, even at room temperature, as reported [24,42]. In our case, using n-type semiconductor material, oxygen vacancies were produced on the surface or in the body of G-ZnO and pure ZnO. Under light irradiation, the conduction band electrons of ZnO were captured by oxygen vacancies. Under the influence of carrier types, the resistance fluctuation of the p-type semiconductor was the opposite of that of the n-type semiconductor.

The process of electron trapping and active oxygen species diffusion produces an electron depletion layer (EDL), and the initial resistance of sensing materials greatly increases [43]. As more electrons are trapped, the depth of the EDL layer becomes thicker, and the resistance of the gas-sensing material becomes higher. As has been analyzed in detail, compared with those of pure ZnO, the amount of oxygen vacancies in G-ZnO is higher, and the amount of active oxygen species is higher, too. Toluene gas reacts with active oxygen species when exposed to it. And G-ZnO illustrates better sensing properties than pure ZnO does. At the same time, electrons return to CB of gas-sensing material, the depth of the EDL and the resistance of gas-sensing material decrease, and the number of free electrons increase.

As discussed above, the outstanding gas-sensing properties of G-ZnO can firstly be explained by the higher amount of oxygen vacancies. Moreover, the existence of graphene can effectively increase the amount of oxygen vacancies in G-ZnO. Secondly, as a good conductive material, graphene increases the conductivity and decreases the baseline resistance of gas sensors. Also, conductive graphene can form Schottky contact with ZnO [44] and reduces the energy barrier between ZnO particles, which is conducive to improving the charge transfer in the sensitive material. In addition, the adsorption of gas molecules on the surface of the sensing material eventually increases by the significant specific surface area of graphene. All of these properties benefit the gas-sensing response.

## 4. Conclusions

In conclusion, graphene-wrapped ZnO nanoparticles composites were successfully fabricated to obtain outstandingly sensitive toluene materials using a straightforward hydrothermal process and heat treatment. Gas-sensing mechanisms were studied in detail. The synergistic effect of the oxygen vacancy defect and the wrapped graphene in the composite sensor were key for the enhanced toluene response. The sensing response of the G-ZnO sensor was 2.7 times that of pure ZnO. Moreover, the G-ZnO sensor showed good selectivity to toluene and worked well under high humidity conditions. Thus, the photo-activated G-ZnO sensor illustrated potential application for the practical detection of toluene in low concentrations under explosive environments.

## Figures and Tables

**Table 1:** Comparison of sensing capabilities in this work with those of toluene sensors reported in the literatures at room temperature.

Sensing Material	Concentration (ppm)	Response	Reference
GR-wrapped ZnO	100	2.70	this work
Ag/Bi_2_O_3_	100	0.89	[33]
ZnO Arrays	6000	0.13	[34]
Pt-SnO_2_	200	0.16	[35]
S-doped MXenes	50	0.80	[36]
3D TiO_2_/GR-CNT	500	0.43	[37]
RGO/PEO	200	0.65	[38]

GR: graphene, CNT: carbon nanotube, RGO: reduced graphene oxide, PEO: polyethylene oxide.

## Data Availability

Data are contained within the article.
